# Novel protein extraction approach using micro-sized chamber for evaluation of proteins eluted from formalin-fixed paraffin-embedded tissue sections

**DOI:** 10.1186/1477-5956-10-19

**Published:** 2012-03-23

**Authors:** Keiichi Hatakeyama, Kanako Wakabayashi-Nakao, Yutaka Aoki, Shun-ichiro Ogura, Ken Yamaguchi, Takashi Nakajima, Taka-Aki Sato, Tohru Mochizuki, Isamu Hayashi

**Affiliations:** 1 Medical Genetics Division, Shizuoka Cancer Center Research Institute, 1007 Shimonagakubo, Nagaizumi-cho, Sunto-gun, Shizuoka 411-8777, Japan; 2 Life Science Research Center, Shimadzu Corporation, 1 Nishinokyo-Kuwabara-cho, Nakagyo-ku, Kyoto 604-8511, Japan; 3 Frontier Research Center, Tokyo Institute of Technology, 4259-B102 Nagatsuta-cho, Midori-ku, Yokohama 226-8051, Japan; 4 Shizuoka Cancer Center Hospital and Research Institute, 1007 Shimonagakubo, Nagaizumi-cho, Sunto-gun, Shizuoka 411-8777, Japan; 5 Pathology Division, Shizuoka Cancer Center Hospital, 1007 Shimonagakubo, Nagaizumi-cho, Sunto-gun, Shizuoka 411-8777, Japan

**Keywords:** Antigen retrieval, Colon adenoma, Colon cancer, FFPE tissue, Gastric cancer, Heat-induced antigen retrieval, Mass spectrometry, Micro-sized chamber

## Abstract

We describe a novel antigen-retrieval method using a micro-sized chamber for mass spectrometry (MS) analysis to identify proteins that are preferentially eluted from formalin-fixed paraffin-embedded (FFPE) samples. This approach revealed that heat-induced antigen retrieval (HIAR) from an FFPE sample fixed on a glass slide not only improves protein identification, but also facilitates preferential elution of protein subsets corresponding to the properties of antigen-retrieval buffers. Our approach may contribute to an understanding of the mechanism of HIAR.

## Background

The standard method for storing clinical specimens in hospital tissue banks is in the form of formalin-fixed paraffin-embedded (FFPE) tissue samples, and these are both abundant and widely available for pathological diagnosis worldwide. Recently, FFPE tissue samples have been subjected to mass spectrometry (MS)-based proteomic analysis [1-4], and several studies in FFPE samples employed heat-induced antigen retrieval (HIAR) to improve protein identification [1,4-12]. These investigations have demonstrated the similarity of protein identification between FFPE and fresh/frozen tissue samples [4,9,12-15]. Thus, FFPE tissues are now considered an alternative to fresh/frozen tissues for protein biomarker discovery.

Although several groups have reported the equivalence of proteomes derived from fresh/frozen and FFPE samples, the mechanism of protein extraction by HIAR is not entirely understood. HIAR was originally developed to improve the sensitivity of immunohistochemical methods [16,17], and is used to unmask epitopes in FFPE samples [18,19]. Recent MS imaging of FFPE tissues suggests that HIAR treatment also increases accessibility of proteins to trypsin [6,20]. However, these studies focused only on the retrieved proteins remaining in the FFPE tissue sections. Chu et al. have shown that HIAR treatment facilitates partial release of macromolecules (polypeptides, nucleic acids, and lipids) from FFPE tissues fixed on a glass slide [21]. In their study, the proteins released from FFPE samples by HIAR were not investigated or characterized by MS analysis.

In this study, we report a novel antigen-retrieval method using a micro-sized chamber for identifying proteins eluted from FFPE tissue samples. FFPE colon cancer tissue sections fixed on a glass slide were treated by HIAR in a fabricated chamber. The method yielded improved protein identification compared to untreated samples, as assessed by MS/MS analysis using a nano-flow LC-ESI linear ion trap (LIT)-TOF mass spectrometer. Our new proteomic approach allows identification and characterization of the proteins eluted from FFPE tissues fixed on the glass slide. We suggest that this antigen-retrieval method can lead to a more in-depth understanding of the mechanisms of HIAR.

## Methods

### Tissue fixation

Tissue samples were prepared from surgically removed human colon and gastric cancer and colon adenoma specimens, in accordance with the standard local therapeutic protocol. Samples (approximately 2 g) were fixed in 10% buffered formalin for 48 h at room temperature, dehydrated with ethyl alcohol, and embedded in paraffin. Paraffin blocks were stored in the dark at room temperature for 2 years.

### HIAR using micro-sized chamber

**Figure 1:**
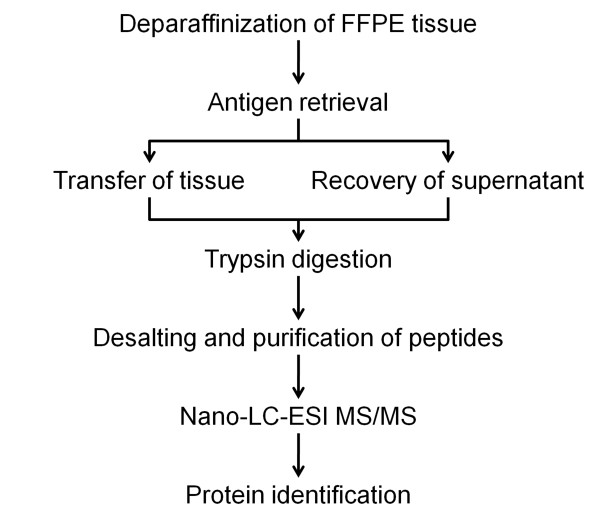
**Workflow for identification of proteins extracted from antigen-retrieved FFPE samples and resulting supernatants**.

**Figure 2:**
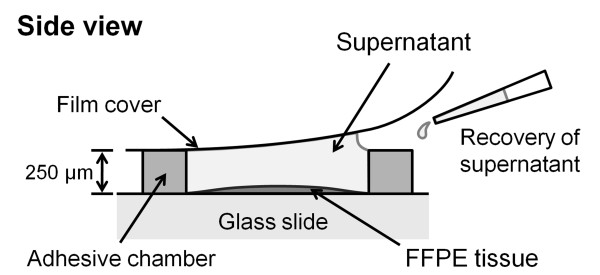
**Illustration of the recovery process for antigen-retrieval buffer in the micro-sized chamber**. The antigen-retrieval buffer was added to the micro-sized chamber adhered to the slide, and then sealed with a film cover. After HIAR, the antigen-retrieval buffer (supernatant) was recovered with a pipette.

The workflow for protein retrieval and identification in FFPE samples is shown in Figure 1. Sections (3-μm thick; approximate area, 15 × 20 mm) were cut and transferred onto a glass slide. Sections were deparaffinized in xylene and rehydrated through a series of alcohol treatment. For HIAR, the deparaffinized sections were incubated in 120 μl of antigen-retrieval buffer (50 mM Tris-HCl, pH 10.0, and 0.1% *n*-octyl-β-D-glucoside) in a micro-sized chamber (Frame-Seal Incubation Chambers for In Situ PCR and Hybridization, 17 mm × 28 mm × 250 μm; Bio-Rad Laboratories, Hercules, CA) or in 30 ml of antigen-retrieval buffer in an incubation vessel (diameter, 66 mm × 101 mm; As One, Osaka, Japan). Chambers were heated to 90-110°C for 10 min in an autoclave. After HIAR in the micro-sized chamber, we collected only the supernatant from the chamber by using a pipette (Figure 2). The collected supernatants were then concentrated using a SpeedVac (Thermo Fisher Scientific, Waltham, MA). After HIAR in the incubation vessel, the collected antigen-retrieval buffer (25-30 ml) was concentrated using an Amicon Ultra-15 Centrifugal Filter Unit with an Ultracel-3 membrane (Millipore, Billerica, MA). We chose this method because precipitated salts from the antigen-retrieval buffer can influence subsequent processes such as trypsinization and MS analysis. The above-described process was repeated twice by using the same paraffin block.

### Trypsin digestion and peptide extraction

FFPE samples with/without HIAR were removed from the glass slide and immersed in 50 mM ammonium bicarbonate. These samples were trypsinized at a final enzyme concentration of 25 μg/ml at 37°C for 18 h. The tryptic digests were twice desalted with ZipTip_μ-C18 _(Millipore). The samples were individually concentrated using the SpeedVac, and reconstituted in 0.1% formic acid.

### LC-MS/MS

MS/MS analysis of samples was carried out on a nano-flow LC-ESI LIT-TOF mass spectrometer (NanoFrontier L; Hitachi High-Technologies, Tokyo, Japan) as previously described [22,23]. The dissolved digests were injected into a 0.05 mm × 150 mm MonoCap for Fast-flow (GL Science, Tokyo, Japan) via a 0.05 mm × 150 mm Monolith Trap C18-50-150 (Hitachi High-Technologies), and the peptides were separated using solvent A (0.1% formic acid and 2% acetonitrile in water)/solvent B (0.1% formic acid and 2% water in acetonitrile) gradient. The gradient profile for solvent B was as follows: 2-40% in 120 min, 95% in 10 min, at 200 nl/min. LC-MS/MS analysis of each digested fraction was conducted in duplicate. The experiment described above was repeated twice.

### Data analysis

Raw LC-ESI data was converted to the various peak list files by NanoFrontier L Data Processing (Hitachi High-Technologies). The peak list files were subsequently applied to MASCOT MS/MS ion search (version: 2.3.01) and X! Tandem http://www.thegpm.org software for protein identification. Upon peptide sequence annotation, the SwissProt database (version: 2010_08) of *Homo sapiens *(human) was used, with the following parameters: enzyme, trypsin; maximum number of missed cleavages, 1; peptide tolerance, 0.2 Da; MS/MS tolerance, 0.2 Da; variable modification, oxidation of methionine and protein N terminus acetylation; and peptide charge, (1+, 2+, and 3+). All identified proteins with MASCOT threshold scores lower than the 95% confidence level and peptide numbers lower than 2 were then removed from the protein list by using Scaffold software (Proteome Software, Portland, OR). Proteins found in both duplicate experiments were counted as the identified proteins.

## Results

### Evaluation of HIAR using micro-sized chamber

**Figure 3:**
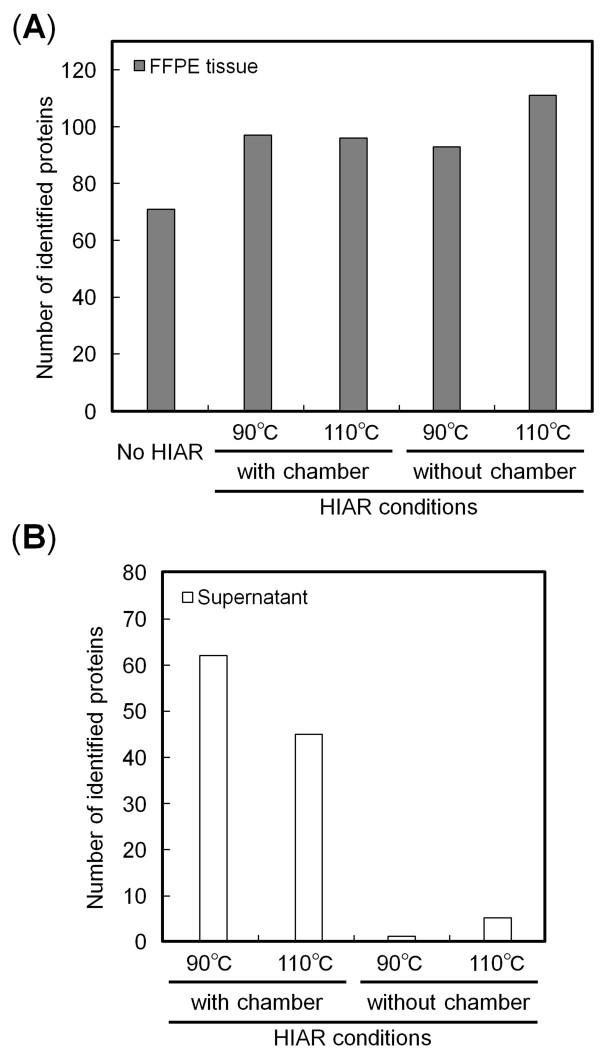
**Number of identified proteins in (A) FFPE samples and (B) supernatants**. FFPE samples underwent HIAR with and without micro-sized chamber in antigen-retrieval buffer at 90°C or 110°C. The number of identified proteins in the trypsinized FFPE sample without HIAR was defined as No HIAR.

To evaluate the feasibility of HIAR using the micro-sized chamber, we compared the number of identified proteins under each sample condition (Figure 3A). The number of proteins identified from FFPE tissues was increased by HIAR compared to that under non-retrieval conditions. No difference in the number of identified proteins was observed between HIAR with or without the micro-sized chamber. These results suggested that protein identification was not influenced by the size of the chamber used for antigen retrieval.

The presence of proteins eluted from FFPE samples was confirmed by MS/MS analysis of the trypsinized supernatants. We identified 62 and 45 proteins in the supernatants after HIAR at 90°C and 110°C, respectively (Figure 3B). In contrast, very few proteins (1 and 5 proteins at 90°C and 110°C, respectively) were detected in the supernatants obtained without the micro-sized chamber. These results indicated that the use of the micro-sized chamber enhanced protein identification from supernatants.

**Figure 4:**
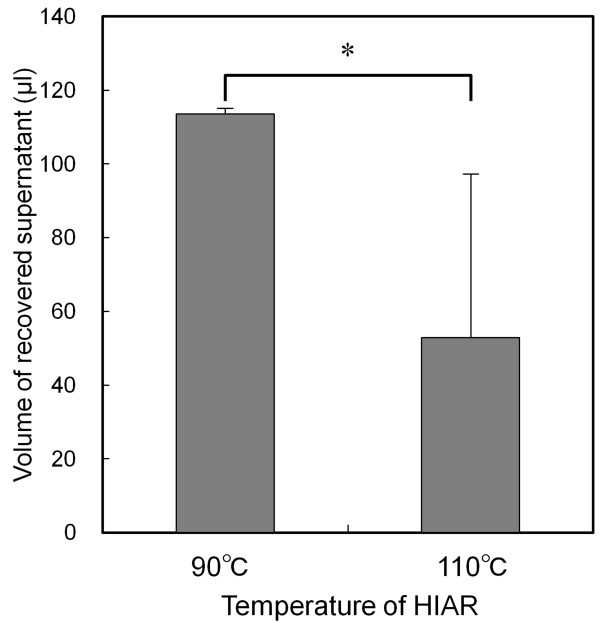
**Amount of supernatant recovered from micro-sized chamber after HIAR**. A total of 120 μl of antigen-retrieval buffer was sealed in the micro-sized chamber. After HIAR, the volume of collected buffer was determined by weight. Data are expressed as mean value ± SD (*n *= 6). Statistical significance (* *P *< 0.01) was evaluated by Student's *t *test.

After HIAR at 110°C by using the micro-sized chamber, a minor leak was observed around the chamber (data not shown). To investigate the cause of the supernatant leak, we measured the volume of recovered supernatant under each retrieval condition without the FFPE sample fixed on the glass slide (Figure 4). A significant decrease in the volume of recovered supernatant was observed at 110°C. On the basis of these results, the HIAR temperature for subsequent experiments was set at 90°C.

To evaluate the efficiency of protein extraction in antigen retrieval with micro-sized chambers, we performed repetitive HIAR treatment against the tissues fixed on the glass slide (Additional file 1: Figure S1). The proportion of proteins identified from the supernatant in repeating treatments was decreased compared to that from the initial HIAR. Nearly all of the eluted proteins in the 2nd-4th HIAR were identical to the proteins identified from the initial HIAR. When identifying proteins from tissues, no significant difference was observed between the first and the final HIAR treatments. The results indicated that complete protein extraction and antigen retrieval were achieved using single HIAR with a micro-sized chamber.

We concluded that our HIAR treatment using the micro-sized chamber allowed protein identification separately in the supernatant and the FFPE samples fixed on the glass slide.

### Characterization of proteins eluted from the FFPE samples

**Figure 5:**
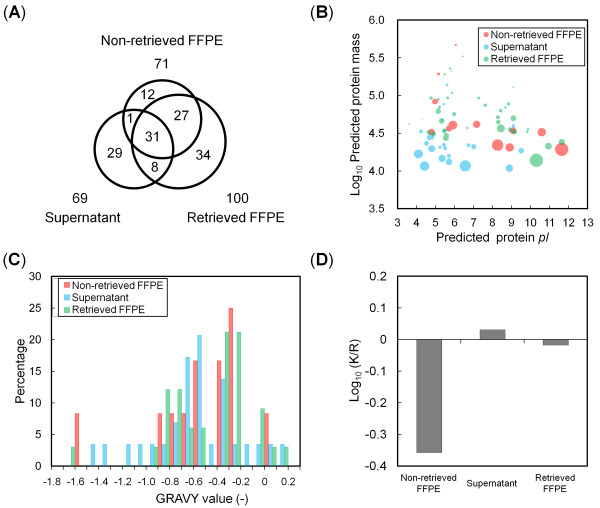
**Characterization of the proteins identified in the non-retrieved and retrieved FFPE sample and the supernatant**. (**A**) Venn diagram comparing the number of proteins identified in the above 3 samples. (**B**) A pseudo 2-D PAGE display of unshared proteins presented in Panel A (size of circle represents the emPAI-determined degree of protein abundance) [24,25]. (**C**) Distribution of the grand average of hydropathy (GRAVY) values of the unshared proteins identified in the above 3 samples. (**D**) Comparison of the log-transformed ratios of C-terminal lysine versus arginine peptides.

The combined proteomic results obtained from 3 samples of different tissues (FFPE tissue sections with and without retrieval, and the eluted supernatant) yielded a total of 142 distinct proteins. These unique proteins are listed in Additional file 2: Table S1. The Venn diagram in Figure 5A shows that 20.4% of the identified proteins were supernatant-specific. Among the 69 proteins identified in the supernatant, 40 proteins were identified in more than one sample. Our analysis required the identification of at least 2 distinct peptides to define a protein hit. Additionally, the proteins identified in only a single experiment were removed from the protein list. Therefore, our results indicated that the unique proteins found in the supernatant were consistently eluted by HIAR treatment from FFPE samples fixed on the glass slide.

Figure 5B shows a pseudo 2-D polyacrylamide gel electrophoresis (PAGE) display of 75 proteins identified in the 3 samples but not shared among them. The figure illustrates the distribution of protein properties, comparing p*I*, molecular weight (MW), and estimated protein content (calculated using the exponentially modified protein abundance index, emPAI) [24,25]. The distribution of proteins identified in the supernatant was shifted toward a lower p*I *and MW. The results suggest that our HIAR approach using micro-sized chambers preferentially promotes elution of low-MW proteins that tend to have a negative charge in the retrieval buffer. Further, the grand average of hydropathy (GRAVY) value [26] of the identified proteins was used to investigate the distribution of hydrophilic proteins (Figure 5C). Most proteins detected in the 3 samples had a negative GRAVY value, suggesting that the identified proteins tended to be hydrophilic. No significant difference was observed between the GRAVY values of the retrieved sample and the supernatant.

Sprung et al. [9] and Azimzadeh et al. [11] observed a preferential detection of peptides with C-terminal arginine over lysine in proteomic analyses of FFPE tryptic digests. We compared the log-transformed ratios of lysine-to-arginine-terminal peptides between the supernatant and the FFPE samples fixed on the glass slide (Figure 5D). A positive log-transformed ratio was observed only in the supernatant. This observed ratio was similar to that previously reported in frozen samples [9]. In contrast, the ratio in FFPE samples with and without HIAR treatment was negative.

## Discussion

Shotgun proteome analyses of FFPE tissue specimens offer the prospect of retrospective biomarker discovery. In these analyses, HIAR treatment is one of the most important techniques for improving protein identification [1,4-12]. Facilitation of the accessibility of trypsin to proteins in FFPE samples via HIAR increases MS-detectable peptides [6,20]. Recently, Chu et al. developed a method for extracting proteins and nucleic acids directly from a fixed section, without destroying tissue morphology, using heat [21]. Their work raised the possibility that HIAR-treated FFPE samples fixed on the substrate may release and lose characteristic sets of proteins depending on the antigen-retrieval buffer. To demonstrate such selective elution, it is desirable to compare protein fragments between the FFPE sample and the buffer (supernatant) after HIAR. To that end, we used a strategy that enabled us to separately collect the supernatant containing MS-detectable peptides and antigen-retrieved FFPE tissues for the identification of eluted proteins.

HIAR for MS analysis has employed various buffers for improving protein identification in FFPE tissues, and these correspond to a variety of conditions for trypsinization and peptide extraction [4-6,8,12]. Many HIAR protocols use an acidic antigen-retrieval buffer followed by buffer exchange and/or washing (including rinsing the tissue) for trypsinization after HIAR. To efficiently trypsinize the retrieved FFPE sample without these additional steps, the use of a basic antigen-retrieval buffer is necessary. The optimal pH range for trypsinization is pH 7-9 [27]. In this study, FFPE tissues fixed on a glass slide were directly treated by HIAR, and then separated from the supernatant without washing. This approach for antigen retrieval is similar to preparation for MS imaging of FFPE tissue, in which HIAR is carried out in basic buffers without washing before trypsinization [6,8]. Moreover, addition of *n*-octyl-β-D-glucoside improves the recovery rate of peptides [28]. Accordingly, we adopted a basic detergent-containing buffer optimized for HIAR of FFPE samples.

To increase efficiency of protein identification in the supernatant, we affixed a micro-sized chamber onto the FFPE samples fixed on the glass slide. The use of the micro-sized chamber significantly reduced the volume of the antigen-retrieval buffer. The small amount of supernatant collected from the micro-sized chamber was concentrated by centrifugal evaporation and trypsinized into one tube. In proteome analysis by MS, reduced handling of protein lysate by use of a single tube increases the efficiency of protein identification [29]. Therefore, our approach is likely to consistently enhance protein identification from the supernatant.

The new approach permitted us to compare the proteins that eluted into the supernatant with those that remained in FFPE tissues. We found that the proportion of low-p*I *and low-MW proteins was notably higher in the supernatant than in the retrieved and non-retrieved FFPE samples, as shown in the pseudo 2-D PAGE display. Our antigen-retrieval buffer was basic (pH 10), resulting in an increase in the negative charge density in the low-p*I *proteins. Negatively charged proteins are thought to be denatured by heating in the macromolecular network structures of the FFPE tissue section [18,30]. Thus, we assume that the denatured low-p*I *and low-MW proteins were preferentially eluted from the network structures into the supernatant under the HIAR conditions used in our study. These findings may indicate that HIAR from FFPE samples fixed on the substrate (e.g., when prepared for MS imaging) facilitates preferential elution of proteins determined by the properties of antigen-retrieval buffers.

The characterized proteins in the pseudo 2-D PAGE display tended to have hydrophilic properties as indicated by the GRAVY value (Figure 5C). No relationship was found between the hydrophilic properties of the proteins and their elution from the FFPE sample. To further investigate the presence of hydrophobic proteins, we mapped transmembrane (TM) domains by using the TM hidden Markov model (TMHMM) algorithm on the website http://www.cbs.dtu.dk/services/TMHMM/[31,32]. Proteins in which TM domains were identified by TMHMM were defined as hydrophobic [33]. Five proteins identified in the FFPE samples, but not in the supernatant, had a theoretical TM domain (see Additional file 2: Table S1), suggesting that TM domain-containing hydrophobic proteins were not eluted into the supernatant by HIAR. Thus, the presence of TM domains in a protein may promote resistance to protein elution from FFPE tissue.

Our HIAR approach showed the disproportionate loss of C-terminal lysine peptides in FFPE samples, consistent with previous results [9,11]. Surprisingly, in the peptides identified in the supernatant, this phenomenon was not observed. In agreement with previous findings [34-37], potential variable methylol (+30 Da) and imine (+12 Da) modifications among all samples did not yield any additional peptides that could be verified through manual spectral evaluation. This result suggested that these modifications were not involved in the shift of the K to R ratio. Therefore, the K to R ratio in the supernatant may indicate that no chemically modified protein fragments were preferentially released from FFPE samples by HIAR.

## Conclusion

Our study describes a new HIAR method with a micro-sized chamber that can efficiently detect and identify proteins eluted from FFPE tissue sections. To our knowledge, this is the first time that a micro-sized chamber has been used to identify and characterize proteins from antigen-retrieved FFPE samples. Using this technique, we show that specific proteins were preferentially released from FFPE samples, an outcome not observed previously by using HIAR.

## Competing interests

The authors declare that they have no competing interests.

## Authors' contributions

KH carried out proteomics experiments, data analysis, and figure preparation. TN and IH conducted sample preparation and evaluated pathological specimens. SO and KH carried out protocol development. KY and TM provided reagents, materials, and analysis tools. KWN, YA, TS, and TM were scientific leads and participated in the design of the study. KWN and KH wrote the manuscript. All authors read and approved the final manuscript.

## Supplementary Material

Additional file 1 **Figure S1**. Relative amount of proteins identified after repetitive HIAR treatments. The graph shows the relative amount of proteins identified after successive rounds of HIAR treatment in a micro-sized chamber. Click here for file

Additional file 2 **Table S1**. List of all identified proteins. The file lists all proteins identified in the FFPE tissues with and without HIAR and in the supernatant. Click here for file
