# Supplementary material for: Novel protein extraction approach using micro-sized chamber for evaluation of proteins eluted from formalin-fixed paraffin-embedded tissue sections
Source: Proteome Sci. 2012 Mar 23;10:19. doi: 10.1186/1477-5956-10-19 (PMC3352043; doi:10.1186/1477-5956-10-19)
Supplement: Additional file 1 — Figure S1. Relative amount of proteins identified after repetitive HIAR treatments. The graph shows the relative amount of proteins identified after successive rounds of HIAR treatment in a micro-sized chamber. [file 1477-5956-10-19-S1.DOC]

**
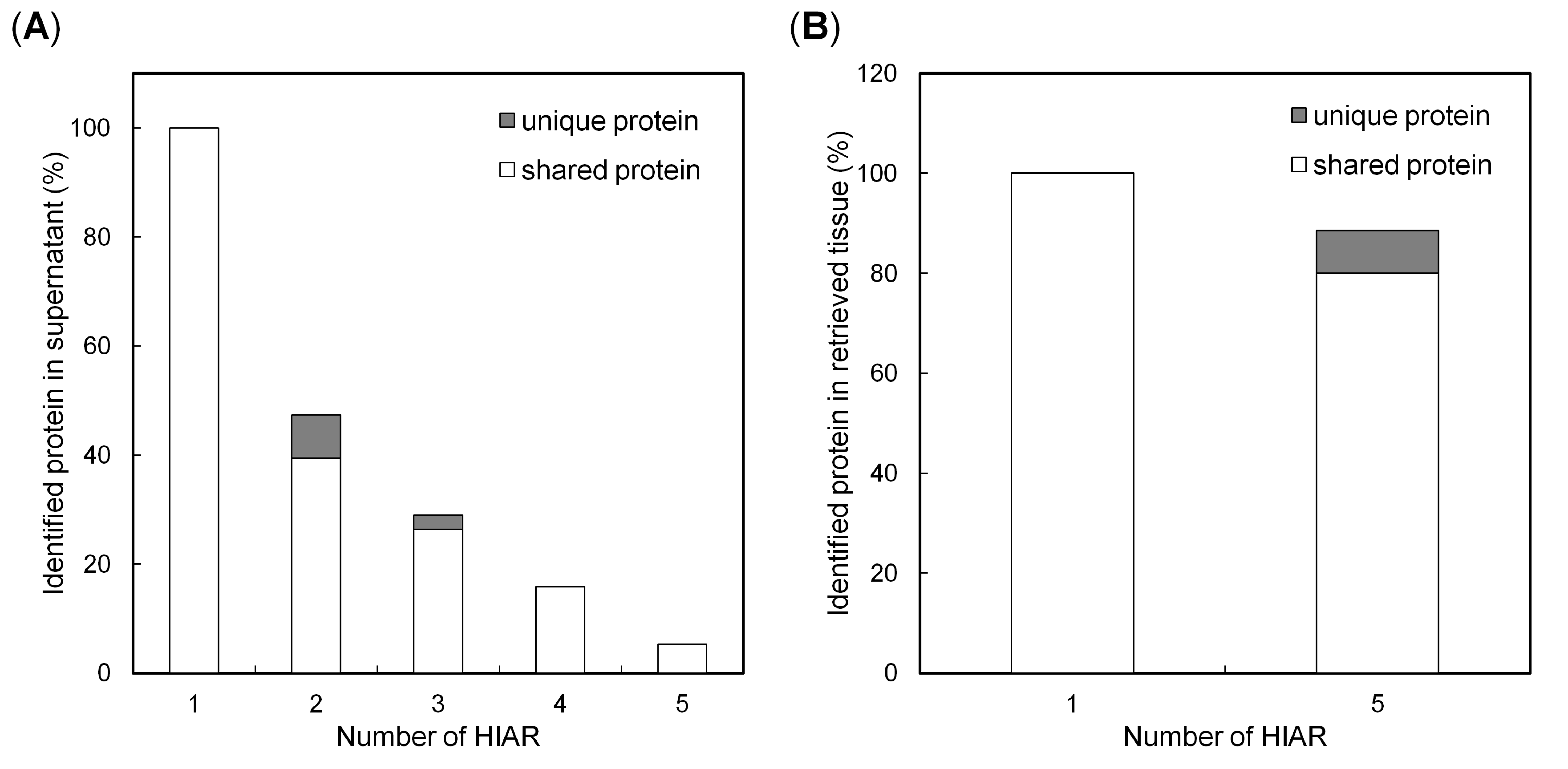
**

**Figure S1.** Relative amount of proteins identified from supernatants (**A**) and tissues (**B**) after repetitive HIAR treatments in a micro-sized chamber.

The relative amount of proteins after the first HIAR treatment was shown as 100%. Unique protein was defined as proteins that cannot be detected after the first HIAR treatment.
