# Supplementary material for: Novel protein extraction approach using micro-sized chamber for evaluation of proteins eluted from formalin-fixed paraffin-embedded tissue sections
Source: Proteome Sci. 2012 Mar 23;10:19. doi: 10.1186/1477-5956-10-19 (PMC3352043; doi:10.1186/1477-5956-10-19)
Supplement: Additional file 2 — Table S1. List of all identified proteins. The file lists all proteins identified in the FFPE tissues with and without HIAR and in the supernatant. [file 1477-5956-10-19-S2.DOC]

**Table S1.** **List of all identified proteins**

| Protein symbol | Protein name | GRAVY  value (-) | Number of TM domains | Number of matched peptides | | |
| --- | --- | --- | --- | --- | --- | --- |
|  |  |  |  | FFPE tissue | | Super-natant |
|  |  |  |  | without HIAR | with HIAR |  |
| CO6A3_HUMAN | Collagen alpha-3(VI) chain precursor | -0.227 | 0 | 17 | 22 | 0 |
| MYH11_HUMAN | Myosin-11 | -0.855 | 0 | 12 | 23 | 0 |
| ACTB_HUMAN | Actin, cytoplasmic 1 | -0.200 | 0 | 12 | 12 | 7 |
| DESM_HUMAN | Desmin | -0.723 | 0 | 14 | 13 | 6 |
| TBB5_HUMAN | Tubulin beta chain | -0.348 | 0 | 6 | 12 | 3 |
| H2B1C_HUMAN | Histone H2B type 1-C/E/F/G/I | -0.738 | 0 | 1 | 3 | 1 |
| FLNA_HUMAN | Filamin-A | -0.317 | 0 | 14 | 18 | 0 |
| K2C8_HUMAN | Keratin, type II cytoskeletal 8 | -0.597 | 0 | 6 | 9 | 5 |
| VIME_HUMAN | Vimentin | -0.823 | 0 | 6 | 9 | 4 |
| 1433Z_HUMAN | 14-3-3 protein zeta/delta | -0.621 | 0 | 5 | 6 | 5 |
| TAGL_HUMAN | Transgelin | -0.584 | 0 | 5 | 5 | 7 |
| TBA1B_HUMAN | Tubulin alpha-1B chain | -0.230 | 0 | 6 | 8 | 5 |
| ACTA_HUMAN | Actin, aortic smooth muscle | -0.233 | 0 | 6 | 5 | 3 |
| ACTN4_HUMAN | Alpha-actinin-4 | -0.637 | 0 | 6 | 6 | 3 |
| ALBU_HUMAN | Serum albumin precursor | -0.354 | 0 | 4 | 7 | 5 |
| HBB_HUMAN | Hemoglobin subunit beta | 0.014 | 0 | 5 | 5 | 4 |
| K1C18_HUMAN | Keratin, type I cytoskeletal 18 | -0.556 | 0 | 3 | 6 | 3 |
| MYL6_HUMAN | Myosin light polypeptide 6 | -0.389 | 0 | 3 | 3 | 5 |
| HSP7C_HUMAN | Heat shock cognate 71-kDa protein | -0.456 | 0 | 4 | 6 | 3 |
| FINC_HUMAN | Fibronectin precursor | -0.543 | 0 | 6 | 5 | 0 |
| LDHA_HUMAN | l-lactate dehydrogenase A chain | -0.006 | 0 | 1 | 4 | 1 |
| KPYM_HUMAN | Pyruvate kinase isozymes M1/M2 | -0.128 | 0 | 5 | 5 | 1 |
| CO6A1_HUMAN | Collagen alpha-1(VI) chain precursor | -0.525 | 0 | 4 | 5 | 0 |
| ANXA5_HUMAN | Annexin A5 | -0.330 | 0 | 4 | 3 | 3 |
| K1C19_HUMAN | Keratin, type I cytoskeletal 19 | -0.532 | 0 | 3 | 7 | 2 |
| HBA_HUMAN | Hemoglobin subunit alpha | 0.048 | 0 | 2 | 3 | 3 |
| H4_HUMAN | Histone H4 | -0.521 | 0 | 7 | 5 | 1 |
| ATPB_HUMAN | ATP synthase subunit beta, mitochondrial precursor | 0.018 | 0 | 3 | 3 | 1 |
| K2C1_HUMAN | Keratin, type II cytoskeletal 1 | -0.626 | 0 | 0 | 12 | 7 |
| TBB2C_HUMAN | Tubulin beta-2C chain | -0.362 | 0 | 3 | 4 | 3 |
| ANXA2_HUMAN | Annexin A2 | -0.524 | 0 | 3 | 5 | 0 |
| MYH9_HUMAN | Myosin-9 | -0.854 | 0 | 3 | 7 | 0 |
| PROF1_HUMAN | Profilin-1 | -0.116 | 0 | 2 | 3 | 2 |
| ENOA_HUMAN | Alpha-enolase | -0.221 | 0 | 3 | 3 | 2 |
| TAGL2_HUMAN | Transgelin-2 | -0.614 | 0 | 2 | 3 | 4 |
| G3P_HUMAN | Glyceraldehyde-3-phosphate dehydrogenase | -0.108 | 0 | 4 | 4 | 2 |
| H2AX_HUMAN | Histone H2A.x | -0.362 | 0 | 3 | 4 | 2 |
| HS90A_HUMAN | Heat shock protein HSP 90-alpha | -0.750 | 0 | 5 | 6 | 3 |
| ROA2_HUMAN | Heterogeneous nuclear ribonucleoproteins A2/B1 | -0.931 | 0 | 4 | 2 | 2 |
| LUM_HUMAN | Lumican | -0.276 | 0 | 1 | 3 | 2 |
| ML12A_HUMAN | Myosin regulatory light chain 12A | -0.827 | 0 | 1 | 3 | 5 |
| ANXA4_HUMAN | Annexin A4 | -0.440 | 0 | 4 | 3 | 1 |
| HSPB1_HUMAN | Heat shock protein beta-1 | -0.567 | 0 | 4 | 3 | 2 |
| PPIA_HUMAN | Peptidyl-prolyl cis-trans isomerase A | -0.316 | 0 | 1 | 4 | 3 |
| TENA_HUMAN | Tenascin | -0.370 | 1 | 4 | 2 | 1 |
| VINC_HUMAN | Vinculin | -0.405 | 0 | 4 | 5 | 0 |
| IGKC_HUMAN | Ig kappa chain C region | -0.500 | 0 | 2 | 2 | 3 |
| TPM2_HUMAN | Tropomyosin beta chain | -1.115 | 0 | 5 | 4 | 1 |
| PLEC_HUMAN | Plectin-1 | -0.665 | 0 | 4 | 5 | 0 |
| POSTN_HUMAN | Periostin precursor | -0.201 | 0 | 1 | 2 | 0 |
| CH60_HUMAN | 60-kDa heat shock protein, mitochondrial precursor | -0.076 | 0 | 3 | 3 | 0 |
| LDHB_HUMAN | l-lactate dehydrogenase B chain | 0.056 | 0 | 2 | 1 | 1 |
| ANXA1_HUMAN | Annexin A1 | -0.419 | 0 | 3 | 5 | 0 |
| TPIS_HUMAN | Triosephosphate isomerase | -0.118 | 0 | 2 | 2 | 2 |
| GSTP1_HUMAN | Glutathione S-transferase P | -0.121 | 0 | 3 | 4 | 2 |
| S10AB_HUMAN | Protein S100-A11 | -0.340 | 0 | 1 | 1 | 2 |
| PDIA6_HUMAN | Protein disulfide-isomerase A6 precursor | -0.275 | 0 | 2 | 3 | 2 |
| LMNA_HUMAN | Lamin-A/C | -0.863 | 0 | 3 | 4 | 0 |
| ATPA_HUMAN | ATP synthase subunit alpha, mitochondrial precursor | -0.067 | 0 | 2 | 2 | 1 |
| NPM_HUMAN | Nucleophosmin | -0.970 | 0 | 0 | 2 | 1 |
| GDIB_HUMAN | Rab GDP dissociation inhibitor beta | -0.332 | 0 | 4 | 4 | 0 |
| PSME2_HUMAN | Proteasome activator complex subunit 2 | -0.355 | 0 | 1 | 2 | 1 |
| 4F2_HUMAN | 4F2 cell-surface antigen heavy chain | -0.147 | 1 | 2 | 2 | 1 |
| IGHG1_HUMAN | Ig gamma-1 chain C region | -0.428 | 0 | 2 | 3 | 1 |
| HNRPK_HUMAN | Heterogeneous nuclear ribonucleoprotein K | -0.705 | 0 | 1 | 2 | 4 |
| HNRPC_HUMAN | Heterogeneous nuclear ribonucleoproteins C1/C2 | -0.977 | 0 | 2 | 2 | 0 |
| PGK1_HUMAN | Phosphoglycerate kinase 1 | -0.078 | 0 | 1 | 3 | 1 |
| CALD1_HUMAN | Caldesmon | -1.693 | 0 | 1 | 3 | 1 |
| MIME_HUMAN | Mimecan | -0.268 | 0 | 1 | 2 | 0 |
| FRIL_HUMAN | Ferritin light chain | -0.520 | 0 | 1 | 1 | 2 |
| CO1A2_HUMAN | Collagen alpha-2(I) chain precursor | -0.648 | 0 | 1 | 1 | 2 |
| RA1L2_HUMAN | Heterogeneous nuclear ribonucleoprotein A1-like 2 | -0.844 | 0 | 2 | 1 | 1 |
| HS90B_HUMAN | Heat shock protein HSP 90-beta | -0.678 | 0 | 2 | 1 | 1 |
| COF1_HUMAN | Cofilin-1 | -0.359 | 0 | 2 | 1 | 3 |
| PDIA3_HUMAN | Protein disulfide-isomerase A3 precursor | -0.506 | 0 | 1 | 3 | 0 |
| CAP1_HUMAN | Adenylyl cyclase-associated protein 1 | -0.352 | 0 | 1 | 2 | 0 |
| EF1A1_HUMAN | Elongation factor 1-alpha 1 | -0.257 | 0 | 0 | 3 | 0 |
| GRP78_HUMAN | 78-kDa glucose-regulated protein precursor | -0.487 | 0 | 2 | 3 | 0 |
| CO1A1_HUMAN | Collagen alpha-1(I) chain precursor | -0.788 | 0 | 0 | 1 | 3 |
| RLA2_HUMAN | 60S acidic ribosomal protein P2 | -0.237 | 0 | 0 | 1 | 4 |
| CSRP1_HUMAN | Cysteine and glycine-rich protein 1 | -0.526 | 0 | 2 | 1 | 1 |
| ENPL_HUMAN | Endoplasmin precursor | -0.713 | 0 | 1 | 3 | 1 |
| PGS1_HUMAN | Biglycan precursor | -0.249 | 0 | 2 | 1 | 0 |
| MYL9_HUMAN | Myosin regulatory light polypeptide 9 | -0.797 | 0 | 0 | 1 | 2 |
| IF4A1_HUMAN | Eukaryotic initiation factor 4A-I | -0.261 | 0 | 1 | 2 | 0 |
| G6PI_HUMAN | Glucose-6-phosphate isomerase | -0.344 | 0 | 1 | 2 | 0 |
| CO6A2_HUMAN | Collagen alpha-2(VI) chain precursor | -0.624 | 0 | 1 | 2 | 1 |
| RL6_HUMAN | 60S ribosomal protein L6 | -0.732 | 0 | 3 | 1 | 0 |
| CLH1_HUMAN | Clathrin heavy chain 1 | -0.243 | 0 | 1 | 3 | 0 |
| UBA1_HUMAN | Ubiquitin-like modifier-activating enzyme 1 | -0.267 | 0 | 2 | 2 | 0 |
| TERA_HUMAN | Transitional endoplasmic reticulum ATPase | -0.348 | 0 | 1 | 2 | 0 |
| 1433S_HUMAN | 14-3-3 protein sigma | -0.599 | 0 | 1 | 1 | 3 |
| HNRPF_HUMAN | Heterogeneous nuclear ribonucleoprotein F | -0.472 | 0 | 0 | 2 | 2 |
| 1433E_HUMAN | 14-3-3 protein epsilon | -0.540 | 0 | 2 | 2 | 1 |
| EF1D_HUMAN | Elongation factor 1-delta | -0.585 | 0 | 1 | 2 | 1 |
| CH10_HUMAN | 10-kDa heat shock protein, mitochondrial | -0.041 | 0 | 0 | 1 | 2 |
| SET_HUMAN | Protein SET | -1.334 | 0 | 0 | 1 | 3 |
| TLN1_HUMAN | Talin-1 | -0.240 | 0 | 2 | 3 | 0 |
| DESP_HUMAN | Desmoplakin | -0.823 | 0 | 1 | 3 | 0 |
| PGBM_HUMAN | Basement membrane-specific heparan sulfate proteoglycan core protein precursor | -0.294 | 0 | 4 | 1 | 0 |
| MYLK_HUMAN | Myosin light chain kinase, smooth muscle | -0.557 | 0 | 1 | 1 | 2 |
| LEG1_HUMAN | Galectin-1 | -0.151 | 0 | 0 | 0 | 2 |
| K1C9_HUMAN | Keratin, type I cytoskeletal 9 | -0.701 | 0 | 0 | 5 | 0 |
| MDHM_HUMAN | Malate dehydrogenase, mitochondrial precursor | 0.143 | 0 | 1 | 2 | 0 |
| EZRI_HUMAN | Ezrin | -0.971 | 0 | 0 | 3 | 0 |
| BGH3_HUMAN | Transforming growth factor-beta-induced protein ig-h3 | -0.085 | 1 | 0 | 2 | 0 |
| PLSL_HUMAN | Plastin-2 | -0.312 | 0 | 0 | 3 | 0 |
| PHB_HUMAN | Prohibitin | 0.024 | 0 | 1 | 3 | 1 |
| SFRS3_HUMAN | Splicing factor, arginine/serine-rich 3 | -1.521 | 0 | 2 | 1 | 1 |
| HNRPQ_HUMAN | Heterogeneous nuclear ribonucleoprotein Q | -0.881 | 0 | 1 | 2 | 0 |
| 1433G_HUMAN | 14-3-3 protein gamma | -0.680 | 0 | 0 | 1 | 2 |
| PRDX1_HUMAN | Peroxiredoxin-1 | -0.265 | 0 | 2 | 1 | 0 |
| COEA1_HUMAN | Collagen alpha-1(XIV) chain precursor | -0.326 | 1 | 2 | 1 | 0 |
| H12_HUMAN | Histone H1.2 | -0.685 | 0 | 1 | 2 | 0 |
| K22E_HUMAN | Keratin, type II cytoskeletal 2 epidermal | -0.471 | 0 | 0 | 2 | 2 |
| RSSA_HUMAN | 40S ribosomal protein SA | -0.309 | 0 | 2 | 1 | 0 |
| CALM_HUMAN | Calmodulin | -0.654 | 0 | 0 | 0 | 2 |
| 1A01_HUMAN | HLA class I histocompatibility antigen, A-1 alpha chain | -0.530 | 1 | 2 | 1 | 1 |
| ROA3_HUMAN | Heterogeneous nuclear ribonucleoprotein A3 | -0.989 | 0 | 1 | 0 | 2 |
| K1C10_HUMAN | Keratin, type I cytoskeletal 10 | -0.624 | 0 | 0 | 2 | 3 |
| PEBP1_HUMAN | Phosphatidylethanolamine-binding protein 1 | -0.652 | 0 | 0 | 1 | 2 |
| 1433T_HUMAN | 14-3-3 protein theta | -0.512 | 0 | 1 | 1 | 2 |
| CNN1_HUMAN | Calponin-1 | -0.746 | 0 | 0 | 2 | 0 |
| MYH14_HUMAN | Myosin-14 | -0.820 | 0 | 1 | 2 | 0 |
| HNRPM_HUMAN | Heterogeneous nuclear ribonucleoprotein M | -0.342 | 0 | 0 | 2 | 0 |
| SODC_HUMAN | Superoxide dismutase [Cu-Zn] | -0.344 | 0 | 0 | 0 | 2 |
| S10A9_HUMAN | Protein S100-A9 | -0.870 | 0 | 0 | 0 | 2 |
| CIRBP_HUMAN | Cold-inducible RNA-binding protein | -1.059 | 0 | 0 | 0 | 3 |
| CEAM5_HUMAN | Carcinoembryonic antigen-related cell adhesion molecule 5 precursor | -0.328 | 0 | 0 | 0 | 2 |
| NACA_HUMAN | Nascent polypeptide-associated complex subunit alpha | -0.655 | 0 | 0 | 0 | 2 |
| YBOX1_HUMAN | Nuclease-sensitive element-binding protein 1 | -1.484 | 0 | 0 | 0 | 2 |
| RL13_HUMAN | 60S ribosomal protein L13 | -0.855 | 0 | 1 | 2 | 0 |
| ARK73_HUMAN | Aflatoxin B1 aldehyde reductase member 3 | -0.292 | 0 | 0 | 2 | 0 |
| IDHP_HUMAN | Isocitrate dehydrogenase [NADP] | -0.397 | 0 | 0 | 5 | 0 |
| PHB2_HUMAN | Prohibitin-2 | -0.258 | 0 | 0 | 3 | 0 |
| CALR_HUMAN | Calreticulin | -1.104 | 0 | 0 | 0 | 2 |
| ITLN1_HUMAN | Intelectin-1 | -0.517 | 0 | 0 | 0 | 3 |
| KCY_HUMAN | UMP-CMP kinase | -0.572 | 0 | 0 | 0 | 2 |
| PEPC_HUMAN | Gastricsin | 0.019 | 0 | 0 | 0 | 3 |
| PERP1_HUMAN | Plasma cell-induced resident endoplasmic reticulum protein | -0.352 | 0 | 0 | 0 | 10 |
| PRDX5_HUMAN | Peroxiredoxin-5 | 0.152 | 0 | 0 | 0 | 7 |
| TXND5_HUMAN | Thioredoxin domain-containing protein 5 | -0.400 | 0 | 0 | 0 | 6 |
